# Retained gastric band port and tube 5 years after gastric band removal and laparoscopic Roux-en-Y gastric bypass: a case report

**DOI:** 10.1186/s12893-018-0448-6

**Published:** 2018-11-22

**Authors:** Ahmad Ghazal, Mourad Niazi, Israa Kannas, Asmaa Alhasan, Hanadi Hawa

**Affiliations:** 1 Surgery Department, Aleppo University Hospital, Aleppo, Syria; 2 Bariatric and laparoscopic surgery, Ohio Clinic, Dubai, UAE; 3 Faculty of Medicine, University of Aleppo, Aleppo, Syria

**Keywords:** Retained gastric band tube, Forgotten foreign body, Gastric band removal, Retained port, Case report

## Abstract

**Background:**

While LAGB has become uncommon in the bariatric surgery practice, band removal with or without revision surgery is still common. Retained postoperative foreign body, of which surgical sponges are the most common, is a rare condition. We report a rare case of retained gastric band port and the attached tube.

**Case presentation:**

A 31-year-old Caucasian female presented to the outpatient clinic, 5 years after her last surgery, complaining of a left upper quadrant abdominal mass over the last 2 years. She had a history of 2 weight loss operations. She had no significant family history nor smoking. CT of the abdomen and pelvis revealed a retained foreign body. On exploration, the port with 10 cm of the connected tube was found and removed through a small incision without laparotomy. The patient made an uneventful recovery.

**Conclusion:**

A bariatric surgeon should be involved in the evaluation of any patient who complains of abdominal pain and/or palpable mass if she/he has a previous weight loss procedure because the bariatric surgeon is fully aware of the possible complications of the bariatric surgeries.

## Background

While LAGB has become uncommon in the bariatric surgery practice, band removal with or without revision surgery is still common. Surgeons performing band removal should be aware of the possible pitfalls and complications of this procedure [1].

Pouch enlargement, band slippage, band erosion, port-site infections and port breakage represent the complications most commonly associated with LAGB [2]. Procedures performed to remove gastric band devices can themselves be subject to complications including retained surgical items [3]. Retained postoperative foreign body, of which surgical sponges are the most common, is a rare condition [4]. We report a rare case of forgotten gastric band port with the attached tube 5 years after gastric band removal and laparoscopic Roux-en-Y gastric bypass.

### Case presentation

A 31-year-old Caucasian female presented to the outpatient clinic five years after her last surgery complaining of a left upper quadrant abdominal mass which is painful on movement, the mass size increased gradually over the last 2 years. She had no fever, diarrhea/constipation or nausea/vomiting.

She had a history of LAGB 10 years ago. Five years later she had a revision surgery due to weight loss failure, the gastric band was removed and laparoscopic Roux-en-Y gastric Bypass was done in the same procedure. Her past medical history included hypothyroidism 13 years ago medically treated by a daily dose of L-thyroxine. She had no significant family history nor smoking.

On examination, the patient was afebrile. The abdomen was soft and non distended, the surgical scars were healed. A 4 × 4 cm, symmetric mass with normal overlying skin was found in the left upper quadrant. This mass was spherical, superficial, tender, firm, mobile and didn’t disappear by compression. There was no bruit or lymphadenopathy.

Laboratory findings including complete blood count, liver function tests, and renal function tests were within normal. The differential diagnosis was port site hernia or retained foreign body.

**Fig. 1:**
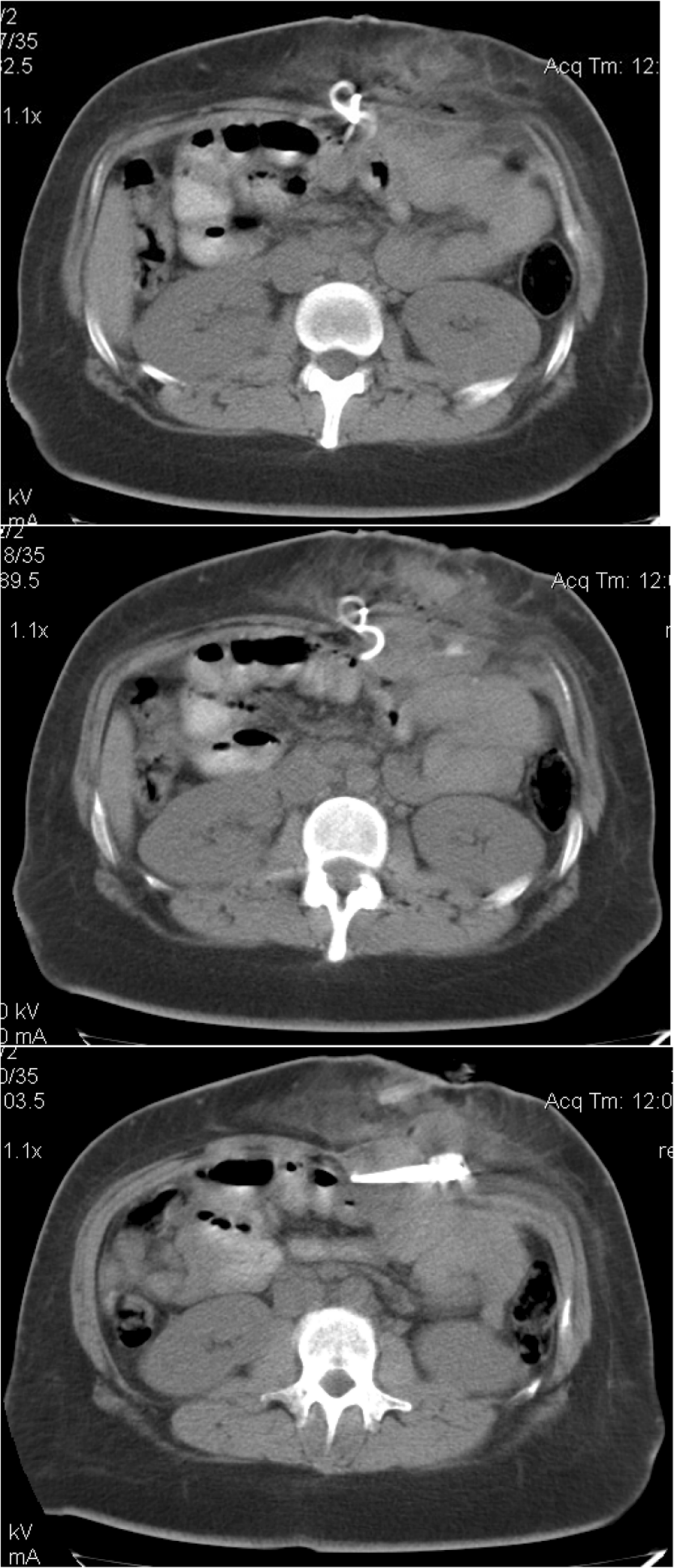
CT of the abdomen and pelvis with oral contrast revealed subcutaneous spherical foreign body (probably the port) with the connected tube extending 10 cm into the abdominal cavity

Further investigations included CT of the abdomen and pelvis with oral contrast revealed subcutaneous spherical foreign body (probably the port) with the connected tube extending 10 cm into the abdominal cavity (Fig. 1). On exploration, the port with 10 cm of the connected tube was found and removed through a small incision without laparotomy. The patient had an uneventful recovery and was discharged on the same postoperative day. The patient expressed her happiness because minimally invasive surgery was done.

### Discussion and conclusion

Gastric band removal is a common procedure [3]. However, forgetting some components is rarely reported in the literature [3].

Cattanach et Teague [3] reported three cases of retained gastric band components after the removal procedures.

Starnes et al. reported a case of retained gastric band tube [1]. Felder et al. reported a case of gastric band as a retained foreign body [5].

Gastric band removal and laparoscopic Roux-en-Y gastric bypass can be a prolonged procedure when there are adhesions. Moreover, the surgical team becomes exhausted at the end of the procedure which may lead to such mistakes. That was the case in our patient where the procedure time was 210 min, the retained foreign body was the port with the connected tube discovered 5 years after gastric band removal and laparoscopic Roux-en-Y gastric bypass.

The main complaint of the patient was a left upper quadrant abdominal mass increased in size over the last 2 years. CT of the abdomen and pelvis could detect a retained foreign body and that was the only necessary diagnostic investigation.

On review, the removal of the gastric band port wasn’t mentioned in the patient’s medical record so we would suggest a checklist to be a part of the surgical notes of gastric band removal procedures.

The possibility of a retained foreign body should be in the differential diagnosis of any postoperative patient who presents with pain, infection, or palpable mass [6].

From a medico-legal standpoint, the surgical team should be fully aware of the consequences of the retained foreign bodies. Therefore, a double check should be done to avoid such cases. Fortunately, the patient didn’t claim against the responsible surgeon for retained gastric band port but that doesn’t apply to every patient.

Detailed history and full clinical examination in addition to a high index of suspicion are mandatory to evaluate patients who have retained foreign body. Hospitals also should develop strategies for LAGB removal procedures to avoid medico-legal issues. Finally, a bariatric surgeon should be involved in the evaluation of any patient who complains of abdominal pain and/or palpable mass if she/he has a previous weight loss procedure because the bariatric surgeon is fully aware of the possible complications of the bariatric surgeries.

## Abbreviations

CT: Computerized tomography
LAGB: Laparoscopic adjustable gastric band
